# Collaboration across the primary and specialist care interface in Early Intervention in Psychosis services: a qualitative study

**DOI:** 10.3399/BJGP.2023.0558

**Published:** 2024-08-20

**Authors:** Michelle Rickett, Tom Kingstone, Veenu Gupta, David Shiers, Paul French, Belinda Lennox, Mike Crawford, Ed Penington, Anna Hedges, Jo Ward, Ryan Williams, Paul A Bateman, Carolyn A Chew-Graham

**Affiliations:** School of Medicine, Keele University, Keele.; School of Medicine, Keele University, Keele; Research and Innovation Department, Midlands Partnership University NHS Foundation Trust, Stafford.; Department of Psychology, University of Durham, Durham; research associate, Department of Nursing and Public Health, Manchester Metropolitan University, Manchester.; School of Medicine, Keele University, Keele; honorary research consultant, Psychosis Research Unit, Greater Manchester Mental Health NHS Trust, Manchester.; Pennine Care NHS Foundation Trust; Department of Nursing and Public Health, Manchester Metropolitan University, Manchester.; Department of Psychiatry, University of Oxford, Oxford.; Division of Psychiatry, Imperial College London, London.; Department of Psychiatry, University of Oxford, Oxford.; Bolton CAMHS, Greater Manchester Mental Health NHS Foundation Trust, Manchester.; Cheshire and Wirral Partnership NHS Foundation Trust, Chester; DHU Healthcare, Derby.; Division of Psychiatry, Imperial College London, London.; Nuffield Department of Primary Care Health Sciences, University of Oxford, Oxford.; School of Medicine, Keele University, Keele.

**Keywords:** continuity of care, Early Intervention in Psychosis, mental illness, primary care, qualitative research, transitional care

## Abstract

**Background:**

People with new psychotic symptoms may be managed in an Early Intervention in Psychosis (EIP) service. They may be discharged back to primary care at the end of their time in an EIP service.

**Aim:**

To explore the role of primary care in supporting people with psychosis in an EIP service.

**Design and setting:**

Qualitative study, within a programme of work to explore the optimum duration of management in an EIP service in England.

**Method:**

Semi-structured interviews were carried out with people in EIP services, carers, GPs, and EIP practitioners between September 2022 and September 2023. Data collection continued until information power was achieved. Data were thematically analysed using principles of constant comparison.

**Results:**

While most service users and carers described their experiences of EIP services as positive, there are issues around access to and discharge from the services. GPs reported difficulties in referring people into EIP services, having little contact with people who are supported by EIP services, and not being included in planning discharge from EIP services to primary care. Service users and carers described challenges at the point of discharge from EIP services to primary care, associated with feelings of abandonment.

**Conclusion:**

This study suggests that GPs should have a role in the support of people in EIP services (in particular, monitoring and managing physical health) and their carers. Inclusion of GPs in managing discharge from EIP services is vital. We suggest that a joint consultation with the service user, their carer (if they wish), along with the EIP care coordinator and GP would make this transition smoother.

## Introduction

People who develop new psychotic symptoms may present to primary care and be referred to, and accepted into, Early Intervention in Psychosis (EIP) services. These are multidisciplinary, community-based mental health teams offering treatment to people who experience a first episode of psychosis. EIP services are designed to intervene to reduce the duration of untreated psychosis, which has been associated with a worse prognosis.^1^ EIP service input has been shown to reduce negative outcomes such as coercive crisis management and hospitalisation under the Mental Health Act.^2^ All service users should have a dedicated EIP care coordinator. Many are also supported by informal caregivers, typically close relatives, including parents, children, partners, and siblings, who can experience lack of support and negative impacts on their own health and wellbeing.^3^

In the UK, EIP services are time limited, offering up to 3 years of treatment. Service users are then discharged either to a community mental health team (CMHT), which offers less intensive contact and interventions, or directly to primary care. Factors influencing discharge to a CMHT include enduring psychoses, referral to EIP from inpatient services, and longer time under EIP care.^4^ There is little guidance around planning and implementation of discharge from EIP to other services,^5^ particularly to primary care,^6^ even though most people are discharged to primary care.^7^ A previous study of transition from EIP services has suggested that better interagency collaboration and service user preparation for discharge, particularly the transition to primary care, is needed.^8^ The need for primary care to ensure that physical needs are met, even when service users are actively engaged with EIP services, has been emphasised previously.^6^

People with psychosis are at risk of developing physical health problems such as diabetes and cardiovascular disease, with the risk of early death.^9^^,^^10^ Physical health monitoring should be carried out at the start of treatment and at least annually, and this may happen in primary care or the EIP service.^11^ Some people may not have appropriate monitoring and management, while others may have duplicate assessments.^12^

**Table:** How this fits in

Early Intervention in Psychosis (EIP) service users may be referred from, and discharged back to, primary care. There is limited research on patients’ and carers’ experience of discharge to primary care from EIP services and little guidance around planning and implementation of discharge. This study explores experiences of EIP care and discharge from the perspectives of service users, carers, and healthcare professionals in EIP services and primary care. It explores the patient journey through EIP services, highlights the lost connection with primary care, and makes recommendations for more collaboration between primary and specialist care, particularly around physical health monitoring and management, which might improve patient experience and outcome.

The aim of this qualitative study was to understand and contextualise the experiences of EIP services and the role of primary care in supporting service users, from the perspectives of recipients and providers of care.

## Method

This qualitative study was part of a larger National Institute for Health and Care Research-funded mixed methods programme (EXTEND: Personalised Care for Early Psychosis), which aims to examine the impact of duration of EIP care on patient outcomes.

Our qualitative methods were underpinned by interpretivism to support exploration of contexts, meanings, and interactions in relation to EIP services from multiple stakeholder perspectives. Semi-structured interviews were used to explore the views and experiences of EIP service users, carers, healthcare professionals from primary and specialist care, managers, and commissioners about EIP services.

Patient and public involvement (PPI) was integral to the study. Our two PPI co-investigators and service user and carer advisory group (EXTEND-InG) co-designed topic guides, public-facing documents, and recruitment strategies. They also contributed to the interpretation of data during analysis meetings with the research team and via email feedback.

### Recruitment of participants

We recruited EIP service users and carers at the time of, or shortly after, discharge from an EIP service (we excluded service users lacking capacity or expressing suicidal ideation), GPs, EIP practitioners and managers, and commissioners of mental health services.

Service users and carers were recruited using both purposive and convenience sampling. Potential participants were identified through mental health trusts (MHTs) across England who reported a mix of high and low duration of treatment. Other participants self-identified by responding to a study flyer shared via social media (X), mental health networks, support groups, and charities. The flyer included a QR code linking to the EXTEND website.

EIP practitioners and managers were identified through participating MHTs. GPs were identified through professional networks and snowballing; we targeted diversity in relation to locality, size, and type of practice.

An information sheet and ‘consent to contact’ form were used. On return of the ‘consent to contact’ form, individuals were contacted by the study researcher (by email or telephone), who checked their eligibility against inclusion criteria, and invited them to participate in an online (Microsoft Teams) or telephone interview, depending on the participant’s preference. The researcher conducted screening calls before interviews with service users and carers to assess capacity and to check eligibility (including age, care under EIP service, and time of discharge). Service users were given the option to be interviewed with a carer.

Consent forms were completed electronically before or at the start of the interview. At the end of the interview, participants were asked if they wished to receive a plain English summary of the research findings and/or related publications. Service users and carers were offered a shopping voucher to recompense them for their time and GPs were reimbursed as per British Medical Association guidance.

### Data generation

Semi-structured interviews were conducted by a female postdoctoral researcher with qualitative research expertise. Interview participants were not given any information about the researcher other than that she was employed by Keele University.

A topic guide supported exploration of the experiences and views of EIP services, duration of care, decision making about discharge, and arrangements for ongoing care. Topic guides were developed with co-investigators and people with lived experience as EIP service users or carers. The guides were modified iteratively alongside data generation and analysis.^13^^,^^14^ Data collection continued until information power was achieved. Information power is an alternative concept to saturation in qualitative research and involves pragmatic judgements based on aims, specificity, theory, dialogue, and analysis.^15^ Interviews were conducted between September 2022 and September 2023.

The interviews were audio-recorded, transcribed by a professional transcribing company, then checked and anonymised by the first author, who also compiled field notes after each interview. Analysis was performed using the qualitative research software NVivo (version 11). Transcripts were not returned to participants for comment and participants were not asked to give feedback on the findings. Data were thematically analysed using principles of constant comparison; this involved the researcher reading and re-reading transcripts, identifying and organising codes, and constructing themes.^16^ A framework approach was used to draw analysis from different participant groups together;^17^ the framework focused on the service user’s journey through EIP services and the complexities around discharge. The first author led the analysis and met regularly with four other research team members, with a range of backgrounds and disciplines, to check data interpretation and agree themes. Members of EXTEND-InG also provided feedback on themes.

## Results

In total, 55 interviews were conducted with 23 EIP practitioners and managers, 13 EIP service users, 10 carers, eight GPs (salaried or partners), and two commissioners. One interview was dyadic (a joint interview with a service user and carer). All EIP practitioners and managers were recruited through MHTs. Eleven service users were recruited through MHTs and two through service user groups. Two carers were recruited through an MHT and eight through social media and online networks. Commissioners and GPs were recruited through professional networks. Twenty-six people replied to our social media service user and carer recruitment flyer but either did not reply to follow-up contacts, or, following a screening call conducted by the first author, were found to be ineligible as they had not been under EIP care, or were thought to be ‘imposter participants’ claiming to be EIP service users or carers but unable to answer questions about EIP care.^18^ Interviews lasted between 25 and 50 minutes.

Key participant characteristics are presented in Tables 1–4. Most service users disclosed physical and/or mental health conditions in addition to the diagnosis of psychosis. A minority of carers (*n* = 3) reported physical and/or mental health diagnoses. Five carers lived with the person they provided care for.

**Table 1.** Service user demographics

**Participant ID**	**Sex**	**Age, years**	**Ethnic background**	**Living circumstances**	**Employment**
SU1	Female	32	White British	Lives with partner and children	Stay-at-home parent
SU2	Female	28	White British	Lives alone	Unemployed
SU3	Female	28	White and Black Caribbean	Lives with partner and child	Part-time employed/student
SU4	Female	26	Mixed/multiple ethnicity	Lives with father	Unemployed
SU5	Female	43	White Polish	Lives with partner and child	Full-time employed
SU6	Female	28	White British	Lives with father	Unemployed
SU7	Female	61	White British	Lives with partner	Unemployed
SU8	Female	57	Black Caribbean	Lives alone	Unemployed
SU9	Male	43	Black British	Lives with partner	Full-time employed
SU10	Female	64	White British	Lives with partner	Unemployed
SU11	Male	25	White British	Lives alone	Full-time employed
SU12	Male	43	White British	Lives with partner and children	Full-time employed
SU13	Male	45	White British	Lives alone	Unemployed

**Table 2.** Carer demographics

**Participant ID**	**Sex**	**Relationship to person with psychosis**	**Age, years**	**Ethnic background**	**Employment**
CAR1	Male	Brother	25	Asian	Full-time employed
CAR2	Male	Friend	26	Black British	Part-time employed
CAR3	Female	Niece	25	Indian British	Part-time employed
CAR4	Female	Mother	65	White British	Retired
CAR5	Male	Husband	59	White British	Full-time employed
CAR6	Male	Grandchild	23	Black British	Part-time employed
CAR7	Female	Mother	59	White British	Part-time employed
CAR8	Male	Partner	65	White British	Retired
CAR9	Female	Former partner	45	Bangladeshi	Full-time employed
CAR10	Female	Mother	45	White British	Part-time employed

**Table 3.** GP background

**Participant ID**	**Sex**	**Years as GP**	**Size of practice**	**Geography**
GP1	Female	10	25k	Inner city
GP2	Male	5	26k	Urban
GP3	Male	5 years as partner	20k	Rural
GP4	Female	25	N/A^a^	N/A^a^
GP5	Male	11 years as partner	14k	Suburban
GP6	Female	19	14k	Urban
GP7	Female	13	10k	Semi-rural
GP8	Female	4	3k	Urban

a *GP4 worked as a locum across several practices in different localities. N/A = not available.*

**Table 4.** Early Intervention in Psychosis practitioner, manager, and commissioner background

**Participant ID**	**Sex**	**Job title**
HCP1	Female	Clinical lead, EIP
HCP2	Female	EIP team manager
HCP3	Female	EIP team manager
HCP4	Female	Care coordinator/mental health nurse
HCP5	Male	Consultant psychiatrist
HCP6	Male	Advanced clinical practitioner/social worker
HCP7	Female	Care coordinator/mental health nurse
HCP8	Female	Community psychiatric nurse
HCP9	Male	Consultant psychiatrist
HCP10	Male	Consultant psychiatrist
HCP11	Female	EIP team manager
HCP12	Male	Psychologist
HCP13	Male	Case manager
HCP14	Male	EIP team manager
HCP15	Female	Clinical nurse specialist
HCP16	Female	EIP team manager
HCP17	Male	Principal psychologist
HCP18	Female	Principal psychologist
HCP19	Male	Community psychiatric nurse
HCP20	Female	Clinical lead, EIP
HCP21	Female	Care coordinator/mental health nurse
HCP22	Male	Community psychiatric nurse
HCP23	Female	Peer support worker
COM1	Male	Commissioner/senior programme manager
COM2	Male	Commissioner/senior programme delivery lead

*EIP = Early Intervention in Psychosis.*

Our findings are presented under four themes: barriers to accessing EIP services; perceived value of EIP services; lost connection with primary care; and discharge planning. Illustrative data are presented to support the analysis along with identifiers.

### Barriers to accessing EIP services

EIP practitioners felt that the initial referral process was straightforward:

*‘So, anyone pretty much can refer directly into EIP, so GPs can come directly even though we have what’s called a Single Point of Access* [SPA] *within the trust, so all GPs usually refer into that SPA service we call it … So if there’s a family member worried about their loved one … and people themselves can refer … so pretty much open-door referral policy, yes.’*

(Healthcare practitioner [HCP]16)

However, GPs described limited knowledge of the criteria for admission to EIP care. Many GPs expressed frustration, a sense of powerlessness at rejected referrals, and concern for patients’ safety and wellbeing:

*‘I referred her to the early intervention, and they did an assessment and basically felt that she wasn’t ill enough to be under their care and just to refer her to the community mental health team because these were just symptoms of anxiety and depression and stress, which I felt was not the route that I would’ve suggested … So it was quite upsetting … I felt really disappointed for this lady because it was a big thing for her to disclose it to me … I was saying, “Well there’s this service called Early Intervention and I think this would be really good”, almost trying to win her around because she was embarrassed to talk about it and then had to come back and be like, “Yeah, and the referral was rejected”, which obviously doesn’t fill her with confidence.’*

(GP1)

### Perceived value of EIP services

EIP staff described the breadth of care offered, and the holistic approach taken, with an emphasis on patient-centred engagement and relationship building being key to the success of the EIP model:

*‘We have the ability to concentrate on engagement and … proper patient-centred care, it’s not just a throwaway line, and we have access to vocational support, to psychology, to social recovery support.’*

(HCP15)

Service users talked about being supported and understood by their care coordinator:

*‘*[Name of care coordinator] *knew so much about me as well, so after three years I didn’t have to tell her who somebody was or anything because she just knew and you’d be like “How’s this person?”, “How’s that person?”, she knew everything about me, so I had no worries there.’*

(Service user [SU]2)

EIP care coordinators were recognised as essential to the success of EIP relationship building:

*‘The relationship and the attachment they have to us is that kind of key bedrock of what helps people.’*

(HCP17)

Service users described how the EIP service had given them better understanding of their condition, which helped them feel less isolated and more in control:

*‘It’s talking to people, people who have really good expertise in psychosis. Because I had no idea what it was. I had all these awesome people around us, like the CPN* [community psychiatric nurse] *and the support worker. I didn’t feel alone, like I could go to them.’*

(SU6)

GPs had limited awareness of the duration of EIP care, what EIP care involves, and the discharge process:

*‘I saw a lady the other day and she was actually discharged from them because she’d been under them for three years, which apparently is the maximum amount of time that they can be under them, which I wasn’t aware of.’*

(GP1)

Some GPs were also unsure about the respective roles of EIP and crisis teams:

*‘I think we’d send them to the crisis team. I mean, I never heard the term EI* [Early Intervention] *until recently*.*’*

(GP6)

Therefore, while supportive relationships were reported to be established between service users and EIP practitioners during the period of EIP care, GPs reported little awareness of the function of EIP services.

### Lost connection with primary care

Service users appreciated the strong supportive relationships they had established with EIP practitioners but reported little to no contact with their GP. As a consequence, service users preferred to speak with EIP practitioners about their physical and mental health:

*‘The GP practice don’t know an awful lot really, I think it’s good that the hospital* [EIP service] *keeps you there quite a few years, I think it’s nice really, you don’t have to deal with your normal GP. Because like I said, I don’t actually know who my GP is.’*

(SU2)

GPs suggested that their role was to refer patients into EIP services (directly or through a crisis team); they would then have little involvement while patients were under EIP care. GPs reported little engagement with EIP care coordinators during the period of EIP care or planning for discharge:

*‘I think the experiences I have had, have given me a reasonable level of trust that those initial stages are handled pretty well in a way that I don’t have to be overly involved as a GP you know, beyond the referral stage.’*

(GP3)

Physical health checks and monitoring for service users were reported to be undertaken by EIP teams:

*‘So, we’re the people meant to be responsible for that. So, the cardiometabolic risk factors, so bloods and BMI* [body mass index] *and blood pressure and smoking,* et cetera*, and particularly regarding the prescribing of antipsychotics. So, we’re responsible for monitoring that. We don’t ask the GPs to do it.’*

(HCP10)

GPs were perceived by EIP practitioners and carers to have responsibility for physical health checks only when EIP services lacked capacity:

*‘Unfortunately, we don’t have a physical health team attached to us at the moment. So, it would be sharing that with the GP and what have you.’*

(HCP22)

*‘I think for that they referred him to his GP because I think they were overloaded with people wanting physical help … and he has been using the same GP for many, many years, so sent him in for the GP and then the GP just conducted physical health evaluations*.*’*

(Carer [CAR] 3)

One GP, working in a small practice, reported that they continued to conduct physical health checks with EIP service users as a way of ‘touching base’ and maintaining a relationship:

*‘We still try and contact the patient and we would still try and have a review in house. That review might be that we say, “Look, we’d like to see you … would you mind coming in just to meet us so we can just touch base with you, see how you’re doing and whether there’s anything else we need to help you with?”’*

(GP8)

In addition, one service user did have an established relationship with the GP, which continued while he was under EIP care.

### Discharge planning

EIP practitioners and managers reported that discharge from EIP was planned well in advance. This was as a result of a recovery-centred approach and long waiting lists for CMHTs:

*‘So, we start thinking about it sometimes six or twelve months ahead and we have a chat about what a person’s needs might be.’*

(HCP10)

*‘I think we work towards discharge from very early on. Although it’s a three-year pathway, we are working … this is our ideal, so we’re working towards discharge. I don’t think we get to the end of the three years and go, “Oh, we need to do some discharge planning.”’*

(HCP14)

For discharge to the CMHT, it was reported that handover meetings were held involving the EIP care coordinator, new CMHT care coordinator, and service user. EIP professionals were aware that this transition could be difficult and required a personalised approach:

*‘So, we would generally try and organise a couple of meetings joint with the case manager from the other team. So, yeah, just sit there and talk, talk about what we’ve done, what the person wants to do, and just so they feel comfortable with their new case manager before we fully discharge them. And we can stretch that out a bit as well if we think someone is particularly anxious about the transition.’*

(HCP13)

Discharge planning to primary care was seen by EIP practitioners as more straightforward, with little contact needed other than via a discharge letter:

*‘I think the liaison is maybe a little less comprehensive than with the adult mental health teams, just because of the nature of the sort of care that’s expected afterwards. So, we would send the GP a letter, to update the GP about the person. There wouldn’t necessarily be a meeting or a discussion; it would be information sharing by letter, really.’*

(HCP11)

*‘If we don’t feel that they need to be in secondary care, we’ll just sort of like discharge them back to their GP … obviously we can just write to the GP and discharge the care back and the consultant will send their letter straight to the GP as well.’*

(HCP7)

EIP practitioners felt that current communication with GPs was sufficient:

*‘We send a letter usually, but if the GP needed to be involved* [in meetings] *we’d invite the GP. It’s very rare GPs come, we do invite them to meetings and professional meetings sometimes talking about somebody’s care, but yeah it would be a letter. And the GP could always contact us.’*

(HCP16)

GPs, however, expressed concerns about EIP discharge communication being limited to letters, which could be received some time after discharge:

*‘Well, there have been instances where I haven’t been aware the person has been discharged. And I can’t remember ever being in a situation where somebody’s deliberately made a point to phone me to say, “your patient’s being discharged”. And often there’s a delay in paperwork so that’s my impression of it. So, I don’t think there is a clear communication to the GP at point of discharge*.*’*

(GP5)

Detailed discharge letters can be an important therapeutic tool for service users and provide a shared narrative of their journey through EIP. GPs, however, reported them to be an unwieldy form of communication:

*‘The mental health discharge letters they can be seven, eight, ten pages long telling us great depth about the full mental state, exam that they’ve done, and things like that. And then just very hard within there to see what the kind of salient points of the case are. You know, the detail is there, but I’m run off my feet in the clinic and I’ve been given this one sort of eight-page letter of someone who’s been in the services a while and they’ve now felt is fit to be discharged.’*

(GP2)

GPs suggested that service users discharged to primary care could still have substantial ongoing support needs. They expressed concern about managing complex antipsychotic medication without specialist support:

*‘The main challenges that I have had have been when the mental health services have wanted to discharge people on, like, say new antipsychotic medications, either medication or depot* [injection]*, but you know, really that should be all under kind of formal shared care agreements between secondary care and primary care, and there’s an increasing push for people to be discharged from the team I think and GPs to take full ownership of the antipsychotic medications.’*

(GP3)

One GP suggested that a transitional approach to discharge could improve communication and joint working between primary care and EIP care, but acknowledged this would require additional time:

*‘Maybe* [service users could] *stay under the early intervention team for longer, but more on an arm’s distance. And so, as GPs, we could kind of see them regularly, but then if we felt we had concerns we could liaise with the early intervention and try and help manage them more remotely, but with their experience, their guidance … I think there would definitely be scope for us to work more with the early intervention team, but it’s just finding the time really*.*’*

(GP1)

Service users and carers expressed worry and uncertainty about discharge from EIP:

*‘I guess I am a bit afraid about what comes next because it does kind of feel like somebody has took the lead off, kind of thing. You won’t be able to talk to the people like you’ve formed a connection with for three years and that’s kind of scary. But you know, I feel like I have the tools to deal with it than I did three years ago.’*

(SU6)

*‘Personally, I’m terrified* [of discharge] *because all my help, I know where to go, I know where to get the help immediately if I need it. Any problems I know what to do and I know his care coordinator so well that I feel comfortable*.*’*

(CAR7)

One service user described how she had relapsed after discharge to primary care, and felt that her concerns about discharge had contributed to this:

*‘I was really sad to be honest, it’s like a weird feeling, it’s been so long just having someone there as like a bit of a comfort blanket and then all of a sudden they’re not going to be there any more and you just think, “Oh no.” Because anything I needed I could call her or text her whenever I wanted. So obviously not having that there was pretty worrying.’*

(SU2)

Most service users described concern about not having continuity with one GP, having to re-tell their story following discharge:

*‘What I have asked is, although I’ve got a named GP at my doctor’s, it has changed where it can be multiple people that you’ve not really seen before. So, I did ask my consultant yesterday if I could … I think for me, it’s about someone who just has a little bit of an idea about what’s gone on previously … but it’s a bit annoying, I think, especially when you are not feeling good mentally, when you’re having to repeat yourself and tell your story time and time again.’*

(SU1)

Carers also felt disconnected from primary care, expressing fear they would not be able to access support for their loved ones after discharge:

*‘My first port of call would be the GP. I wouldn’t know who I would be speaking to there … and I would feel like we have to go back to square one again and start again. And so I would be doing that out of desperation with no confidence that there would be an appropriate reaction*.*’*

(CAR8)

Although complex medication regimens were of concern to GPs, medication management could offer a mechanism for service users to build a relationship with their GP:

*‘One thing that was really helpful and has now stopped is she was having her depot at the GP and she was absolutely happy to go. She felt comfortable with the* [practice] *nurses, and they had a chat. It wasn’t the same one I don’t think, and she’d say, “Oh, it was* [Name] *today and we talked about this.” So that did build a certain relationship with the practice.’*

(CAR4)

## Discussion

### Summary

This qualitative study illustrates the tensions across the primary and specialist care interface for EIP services in England. GPs and service users reported difficulties in accessing EIP services and challenges at the point of discharge. As referrals to EIP services are often made at crisis point, service users may not have been known to their GP before referral, and there may not be a pre-existing relationship to pick up on discharge from the service. We report that service users under an EIP service may have little contact with a GP. Further, physical health monitoring may not be done in primary care.

Experiences while in EIP services were described as positive by most service users and carers in this study, with service users highlighting the benefits of regular and continuing support from care coordinators. Discharge to CMHTs was reported to be well planned and proactive, although sometimes impeded by long waiting times for admission to CMHTs. This contrasted with discharge to primary care being seen by EIP professionals as needing no more than a letter to the GP informing them of the discharge. Both service users and carers described feelings of abandonment when discharged to general practice, often to a GP they did not know.

### Strengths and limitations

This study explores experiences of EIP services from multiple perspectives and illustrates the complexity of the patient journey through these services. We recruited a diverse sample and analysis was conducted by a multidisciplinary team.

There are limitations to this study. Of the 13 service user participants, only four were male, yet most people in EIP services are male.^19^ Seven of the ten carer participants were caring for male service users, which gave additional insight. Four service users were non-White despite attempts to recruit an ethnically diverse sample through MHTs and social media. Service user participants were all stable in terms of their mental health at the point of interview. Carers shared experiences of service users who were unwell, to support a broader understanding. Most service user participants had become unwell before the COVID-19 pandemic; more recent experiences of a new psychotic illness may reveal important differences. We gave service users and carers the opportunity to be interviewed together if they preferred but only one dyadic interview was completed.

### Comparison with existing literature

Our study adds to the very limited research on patients’ and carers’ experience of discharge to primary care from EIP services, even though this is the most common outcome following EIP service input.^6^^,^^7^ Service users value the continuity of care provided by EIP services — particularly the opportunity to build a therapeutic relationship with a named care coordinator — which is consistent with previous literature.^20^ One study has shown that continuity of care in EIP services results in higher service user satisfaction and better health outcomes.^5^ Similarly, continuity for people with severe mental illness (SMI) leads to better health outcomes, reduced hospital admission, and is more cost-effective.^21^

Supporting the physical health needs of people with SMI is one of the five clinical priorities of the CORE20PLUS5 approach,^22^ and is particularly urgent given the 15-year mortality gap between people with SMI and the general population.^23^ The Positive Cardiometabolic Health Resource (Lester update) emphasised the importance of physical health monitoring from the earliest stages of a psychotic illness, particularly when antipsychotic medication is prescribed,^11^ as the risk of weight gain in the first 12 months requires early intervention.^9^

The lack of contact with primary care while people are managed by EIP services reduces opportunities for service users and carers to establish relationships with general practice and engage with physical health monitoring.^24^

### Implications for practice

We suggest it would be beneficial for closer connections to be maintained between patients in EIP services and their registered general practice — there are various ways to achieve this. This could be a ‘named GP’ to take over prescribing on discharge and/or a healthcare assistant or practice nurse tasked with conducting the physical health check.^25^ Mental health practitioners, embedded in primary care teams (as part of the Additional Roles Reimbursement Scheme),^26^ could maintain contact with service users under EIP care and liaise with care coordinators, GPs, and carers to plan discharge. A joint consultation between the service user, their carer (if they wish), care coordinators, and primary care clinicians would support effective transitions. Supporting the service user and carer to understand when and how to access primary care would be valuable, particularly if the person were to become unwell again. Closer connections with primary care would also promote relationship-based care, which is already a hallmark of the care that people receive in EIP services. Our findings could be used to support the education and training of primary and specialist care practitioners, and commissioners.

We suggest that current discharge letters are not used effectively to communicate with primary care. A standard structured discharge letter should be jointly developed, which summarises the key points of importance for primary care clinicians.^27^

Primary care also has a role in supporting carers,^3^ so maintaining contact with, and offering support to, the carer of a person being managed by EIP services is vital.
